# *In vitro* differentiated human CD4^+^ T cells produce hepatocyte growth factor

**DOI:** 10.3389/fimmu.2023.1210836

**Published:** 2023-07-13

**Authors:** Shayne Lavondua Ford, Terkild Brink Buus, Claudia Nastasi, Carsten Geisler, Charlotte Menné Bonefeld, Niels Ødum, Anders Woetmann

**Affiliations:** ^1^ The LEO Foundation Skin Immunology Research Center, Department of Immunology and Microbiology, Faculty of Health and Medical Sciences, University of Copenhagen, Copenhagen, Denmark; ^2^ Immunopharmacology Unit, Department of Oncology, Mario Negri Pharmacological Research Institute (Istituto di Ricovero e Cura a Carattere Scientifico (IRCCS)), Milan, Italy

**Keywords:** T cell, HGF, c-Met, hepatocyte growth factor, cytokine, growth factor, sequencing

## Abstract

Differentiation of naive CD4^+^ T cells into effector T cells is a dynamic process in which the cells are polarized into T helper (Th) subsets. The subsets largely consist of four fundamental categories: Th1, Th2, Th17, and regulatory T cells. We show that human memory CD4^+^ T cells can produce hepatocyte growth factor (HGF), a pleiotropic cytokine which can affect several tissue types through signaling by its receptor, c-Met. *In vitro* differentiation of T cells into Th-like subsets revealed that HGF producing T cells increase under Th1 conditions. Enrichment of HGF producing cells was possible by targeting cells with surface CD30 expression, a marker discovered through single-cell RNA-sequencing. Furthermore, pharmacological inhibition of PI3K or mTOR was found to inhibit HGF mRNA and protein, while an Akt inhibitor was found to increase these levels. The findings suggest that HGF producing T cells could play a role in disease where Th1 are present.

## 1 Introduction

A fascinating aspect of naive CD4^+^ T cell biology is that the cells can differentiate into specialized subsets that are suited for the resolution of specific categories of inflammation (1). The first of these subsets, Th1 and Th2, were identified from mouse T helper (Th) cell clones, which were found to produce different cytokines when stimulated. Th1 produced IFN-γ, GM-CSF, and IL-3, while Th2 produced IL-3, IL-4, and, at the time, additional unknown factors (2). The characteristic cytokine profile of Th1 and Th2 cells has been well defined since then, as well as the factors which influence the direction of the differentiation process, and the category of infection the cells are suited to resolve. For Th1, IL-12 drives differentiation, and the main characteristic cytokine produced is IFN-γ (3). Whereas for Th2, IL-4 drives differentiation, and the characteristic cytokines produced include IL-4, IL-5, and IL-13 (3). Several additional subsets have been defined, which include Th17 and regulatory T cells (4). Together, these Th subsets represent the capacity of the immune system to generate suitable response to myriad pathogenic threats. For intracellular bacteria and viruses, Th1 enhance clearance by activating macrophages through CD40L and by producing IFN-γ (5–8). For large extracellular parasites, Th2 can orchestrate granulocyte and humoral response through IL-4, IL-5, and IL-13 (6, 9). In the case of the microbiome of the gut, Th17 cells restrain pathogenic invasion through the intestinal lumen by attracting neutrophils and activating epithelial and stromal cells, while T regulatory cells (Treg) suppress inordinate response against commensal bacteria and food, with the balance of either cell type controlled through the environmental signals received by mucosal dendritic cells (10–13).

Hepatocyte growth factor (HGF) was discovered as a mitogenic factor for hepatocytes during research of liver regeneration in rats (14, 15). HGF was later purified from human serum, shown to be mainly produced by cells of mesenchymal origin, and was identified as a factor promoting motility and invasiveness in epithelial cells (16–19). The receptor for HGF is mesenchymal-epithelial transition factor (c-Met), a receptor tyrosine kinase mainly expressed by epithelial cell types, which can activate a broad array of signal transduction pathways including MAPK, PI3K-Akt-mTOR, STAT3, and NF-κB (20, 21). Conceptually, the cellular effects of c-Met signaling have been described as activating motility, growth, and survival; collectively dubbed ‘invasive growth’ (22). Reports of T cells producing HGF are limited, though there is one published instance where production of HGF protein by a birch antigen specific human CD4^+^ T cell line was shown (23). In this study, we report that *in vitro* differentiated human CD4^+^ T cells can produce HGF.

We found that *in vitro* differentiated CD4^+^ T cells that had been treated with a Th1 cocktail were the most positive for HGF, and the proportion of positive cells can be increased by enrichment based on CD30 surface protein expression. We studied signal transduction using small molecule kinase inhibitors and a T cell line, PSO-2, that spontaneously produces HGF, which indicated that the driver of HGF production is downstream of mTOR. Conversely, Akt inhibition greatly increased HGF in both the PSO-2 cell line as well as in primary T cells. Together, we show that HGF is expressed by human T cells, particularly under Th1 polarizing conditions, and consequently may play a role in Th1 driven disease.

## 2 Materials and methods

### 2.1 RT-qPCR

RNA was extracted from cell pellets using the RNeasy Mini Kit according to the manufacturer’s instructions, with the modification of two additional RPE wash steps (Qiagen, 74106). The RNA was quantified using a NanoDrop 2000 (ThermoFisher, P/N ND-2000). Equal quantities of RNA (normalized across a given experiment) were used to synthesize cDNA using a High-Capacity cDNA Reverse Transcription Kit, according to the manufacturer’s instructions (Applied Biosystems, P/N 4368814). TaqMan gene expression assays mixed with Light Cycler 480 Probes Master (Roche, P/N 04887301001) were used to quantify mRNA levels using a Light Cycler 480 real-time PCR system, as per the manufacturer’s instructions (Roche, P/N 05015243001). Fold change was calculated using the ΔΔCT-Method. HGF gene expression was not detected in certain cell populations in several of the experiments, and in such cases an arbitrary CT value of 42 was manually entered. In cases where sample groups had no detectable HGF and CT value(s) of 42 were manually entered, statistical tests to compare those groups to other groups were not conducted. Therefore it was not possible to assess if a statistically significant difference between such groups existed. HGF expression was qualified based on comparison to the highest expression level of this gene by *in vitro* differentiated Th1-like cells, which peaked at 35.54 CT value after 5 days of *in vitro* differentiation into Th1-like cells. We considered this value to be a high expression level since Th1-like cells were positive for HGF mRNA and produced HGF protein. The following TaqMan probes were used: POLR2A Hs00172187_m1, G6PD Hs00166169_m1, HGF Hs00300159_m1, TBX21 Hs00894392_m1, IFNG Hs00989291_m1, TNFRSF8 Hs00174277_m1, TNF Hs01113624_g1, MRC1 Hs80267207_m1, CD274 Hs01125301_m1.

### 2.2 Cell culture

PSO-2 cells were cultured in RPMI-1640 (Sigma-Aldrich, P/N R2405) supplemented with 10% human serum (HS) (heat-inactivated, Rigshospitalet, Denmark), 1% penicillin/streptomycin (P/S) (Sigma-Aldrich, P/N P0781), 1000 IU/mL IL-2 (Novartis Proleukin, P/N CLB-P-476-800-19438DK), and 25 ng/mL IL-4 (Leinco, P/N I-186) (24). MF2059 cells were cultured in RPMI-1640 supplemented with 10% fetal bovine serum (heat-inactivated, Biological Industries, P/N 04-007-1A) and 1% penicillin/streptomycin (25). Base media for primary T cell experiments was RPMI-1640 supplemented with 10% human serum, 1% P/S, 25 mM HEPES (Sigma-Aldrich, P/N 83264), 1x GlutaMAX (Gibco, P/N 35050-038), and 1000 IU/mL IL-2. Media composition inspired by (26).

### 2.3 Purification of peripheral blood mononuclear cells from buffy coats and specific cell types from PBMCs

Informed, written consent was obtained from the blood donors at the Department of Clinical Immunology, University Hospital Rigshospitalet, Copenhagen, and donated material was used without the possibility to identify case specific information. The ethical committee, Region H, Capital Region of Denmark, approved the use of the buffy coats for research that was carried out in accordance with the approved guidelines. PBMCs were purified from healthy donor buffy coats by density gradient centrifugation with Lymphoprep (StemCell Technologies, P/N 07801) and SepMate tubes (StemCell Technologies, P/N 85450). Red blood cells were lysed using RBC lysis buffer (Miltenyi Biotec, P/N 130-094-183) or sterile Milli-Q water. Naive T cells were isolated from PBMCs using EasySep Human Naive CD4^+^ T Cell Isolation Kit (StemCell Technologies, P/N 19555, magnets P/N 18001) following the manufacturer’s instructions. Memory CD4^+^CD45RO^+^ T cells were enriched from PBMCs using MagniSort Human CD4 Memory T cell Enrichment Kit (Invitrogen, P/N 8804-6813-74) according to the manufacturer’s instructions. Monocytes were isolated from PBMCs using EasySep Human Monocyte Isolation Kit (StemCell Technologies, P/N 19359) according to the manufacturer’s instructions. CD4 and CD8 T cells were isolated from PBMCs using EasySep Human CD4^+^ T Cell Isolation Kit (StemCell Technologies, P/N 17952) or EasySep Human CD8^+^ T Cell Isolation Kit (StemCell Technologies, P/N 19053) following the manufacturer’s instructions.

### 2.4 T helper cell differentiation

For Th-like differentiation, freshly isolated naive CD4^+^ T cells were resuspended in base media (see above) supplemented with the appropriate cocktail listed below. In addition, CD3/CD28 beads (Gibco 1132D) were added at a ratio of 2 beads per 5 cells. The cells were cultured undisturbed in this media for 5 days in 6 well plates (Corning, P/N 3516). Th1: 4 µg/mL anti-IL-4 (PeproTech, P/N 500-M04), 10 ng/mL IL-12 (R&D Systems, P/N 219-IL-005) (27). Th2: 12.4 ng/mL IL-4 (Leinco, P/N I-186), 4 µg/mL anti-IFN-γ (PeproTech, P/N 500-M90), 5 µg/mL anti-IL10 (PeproTech, P/N 500-M86) (28). Th17: 4 µg/mL anti-IL-4 (PeproTech, P/N 500-M04), 30 ng/mL IL-6 (PeproTech, P/N 200-06), 20 ng/mL IL-23 (PeproTech, P/N 200-23), 5 ng/mL TGF-β1 (PeproTech, P/N 100-21), 4 µg/mL anti-IFN-γ (PeproTech, P/N 500-M90) (27). Treg: 100 ng/mL Rapamycin (Tocris, P/N 1292), 100 nM all-*trans* retinoic acid (Sigma-Aldrich-Aldrich, P/N R2625), 5 ng/mL TGF-β1 (PeproTech, P/N 100-21) (27). Note: an earlier version of this differentiation method was used to generate the cells that were used in few ELISpots. In the previous version, X-VIVO 15 (Lonza, P/N BE02-060F) was used for the base media instead of RPMI-1640, and the concentration of human serum was 5% instead of 10%.

### 2.5 Monocyte and macrophage differentiation

For monocyte differentiation into macrophages, 2x10^6^ freshly isolated monocytes were resuspended in 5 mL base media (without IL-2) supplemented with either 10 ng/mL M-CSF (PeproTech, P/N 300-25) or 10 ng/mL GM-CSF (PeproTech, P/N 300-03) (29). The cells were cultured undisturbed in this media for 5 days in untreated 6 well plates (Corning, P/N 3736). After 5 days the media was removed from the plate, adherent cells were washed with sterile PBS, and then detachment was performed by adding TrypLE Express Enzyme (Gibco, P/N 12605028) and incubating the cells in an incubator for 10 minutes (37°C, 5% CO_2_, humidified). For M0, M1 or M2 macrophage differentiation, macrophages in base media (without IL-2) were seeded into 24 well plates (Corning, P/N 3524). For M1 differentiation 20 ng/mL IFN-γ (PeproTech, P/N 300-02) and 100 ng/mL lipopolysaccharides (LPS) (Sigma-Aldrich, P/N L4524) were added to the media (30). For M2 differentiation 10 ng/mL IL-4 (PeproTech, P/N 200-04) and 10 ng/mL IL-13 (PeproTech, P/N 200-13) were added to the media (30). For M0, nothing was added to the media. The differentiation period was 24 hours. In some cases, 50 ng/mL HGF was added during or after M1/M2 differentiation (PeproTech, P/N 100-39H). For the macrophage and T cell co-culture experiment, monocytes that had been differentiated into macrophages with M-CSF were seeded into 24 well plates at a concentration of 5.5x10^4^ cells per well and treated with either an M1 or M2 differentiation cocktail for 24 hours. After this period, the media was removed, cells washed with sterile PBS, and then 1 mL of base media containing 1x10^6^ donor matched differentiated CD4^+^ T cells and CD3/CD28 beads (ratio of 1 bead per 8 T cells) was added to the wells. After 24 hours, the T cells and beads were resuspended in the wells and then collected. The well was washed 5 times with sterile PBS, and then the adherent cells in the well were lysed with Buffer RLT (Qiagen, P/N 79216), lysate collected and then stored at -80°C. Beads were removed from the supernatant collected earlier, T cells were pelleted, supernatant discarded, and then the cell pellets were stored at -80°C.

### 2.6 Enzyme-linked immunosorbent assay

The concentration of HGF protein in supernatants was assayed with the Human HGF DuoSet ELISA (R&D systems, P/N DY294), DuoSet ELISA Ancillary Reagent Kit 2 (R&D Systems, P/N DY008B), and NUNC MaxiSorp ELISA plates (Merck, P/N M9410), according to the manufacturer’s instructions.

### 2.7 Small molecule kinase inhibitors

PI828 (Tocris, P/N 2814), PP242 (Tocris, P/N 4257), API-2 (triciribine) (Tocris, P/N 2151), Rapamycin (Tocris, P/N 1292).

### 2.8 Lactate dehydrogenase assay

Detection of the cytoplasmic enzyme lactate dehydrogenase in the supernatant of cell cultures was used as a proxy for evaluating the toxicity of small molecule kinase inhibitors. LDH Cytotoxicity Detection Kit was used as per the manufacturer’s instructions (Takara Bio, P/N MK401).

### 2.9 Enzyme-linked immunosorbent spot assay

The HGF ELISpot protocol is based on MABTECH’s “Human IFN-γ ELISpot^BASIC^” protocol, but with different capture, detection, and substrate reagents, as well as a longer substrate development time. The matched antibody pair that is used in the R&D Systems HGF ELISA was used at higher concentrations for the capture and detection of HGF protein in the HGF ELISpot. The capture antibody was used at a concentration of 1 µg/well (R&D Systems, P/N MAB694), and the detection antibody was used at a concentration of 0.1 µg/well (R&D Systems, P/N BAF294). Streptavidin alkaline phosphatase conjugate (1:1000 dilution) (MABTECH, P/N 3310-10) and BCIP/NBT-plus substrate (MABTECH, P/N 3650-10) were used for detection of the biotinylated detection antibody. The ELISpot was conducted with plates that had PVDF membranes (Merk, P/N MSIPS4W10). The protocol was as follows: The following steps were performed in a sterile bench. The PVDF plate was treated with 15 µL 35% alcohol per well for 1 minute and then washed 5 times with sterile PBS (when emptying the plate, gentle motions were used to avoid imprinting the underdrain onto membranes and/or causing a crease to form in the center of the membranes). 100 µL/well capture antibody diluted in PBS was added to each well, and then the plate was sealed and incubated overnight at 2-8°C. The next day, the coating solution was removed by topping up the wells with sterile PBS and then gently tapping the plate onto paper towels. The wells were washed 5 times with 200 µL/well PBS. 200 µL/well base media without HS (see cell culture section) was added to the wells, and then the plate was incubated (37°C, 5% CO_2_, humidified) for 1 hour. During this time, cell suspensions and treatments were prepared and dispensed into a 96 well round bottom plate (T cells 5x10^5^ cells/well, and cell lines 1x10^5^ cells/well, 250 µL volume, base media without HS). After the 1-hour period, the media was removed from the ELISpot plate and cell suspensions transferred from the 96 well plate to the ELISpot plate. The plate was then put into a level incubator (37°C, 5% CO_2_, humidified) undisturbed for 24 hours. After 24 hours, cells were recovered from the ELISpot (cell recovery: cells were resuspended without touching the well membranes with pipette tips, and then as much volume as possible was transferred to a 96 well plate. 100 µL PBS was used to dilute the remaining volume in the ELISpot wells, and then as much volume as possible was transferred to the 96 well plate). The remainder of the protocol was conducted outside of a sterile bench. The ELISpot plate was washed 5 times with PBS. The detection antibody was diluted in 1% BSA PBS, filtered using a 0.2 µm syringe filter (Fisherbrand, P/N 15206869), and then dispensed (100 µL/well) into the wells and left to incubate at room temperature for 2 hours. After 2 hours, the plate was washed 5 times with PBS. Strep-ALP was diluted 1:1000 in 0.5% BSA PBS, 100 µL/well was dispensed, and the plate was left to incubate for 1 hour at room temperature. After 1 hour, the plate was washed 5 times with PBS, and then 100 µL/well 0.45 µm syringe filtered (Fisherbrand, P/N 15216869) BCIP/NBT-plus substrate was added. After 1 hour of room temperature development time, the plate was washed with DI water. The underdrain was removed to facilitate washing and faster drying. Spots were counted using either CTL ImmunoSpot 5.0.9 software (**Figures 8J**, **S5E**) or FIJI(ImageJ) Weka segmentation followed by particle count. For the counts in **Figure 2C** (this graph is summary data from 3 ELISpot plates), the software was trained to count dark spots based on PSO-2 and Th1 wells, and to ignore light artifacts and variations in background that occur, for example in the MF2059 wells (e.g., **Supplemental Figure 1A**, row A: 2059 versus row B: PSO-2). Once the classification model that gave an accurate count was generated for the first plate, the same model was used to generate the counts for the other plates. Note: an earlier version of the HGF ELISpot used 1.5 µg/well capture antibody and 1x10^6^ T cells/well and the ELISpot in **Figures 8J** and **S5E** was run with that version.

### 2.10 Single-cell RNA sequencing

First scRNA-sequencing run (Th1-like): Th1-like cells were generated from a single donor as described in the previous sections. After 5 days of differentiation, the cells were washed and then 1x10^6^ cells plated in base media with and without CD3/CD28 beads (ratio 1 bead to 8 cells). After 24 hours, the cells were harvested and 2x10^5^ cells from each condition were stained with cell hashing antibodies targeting β2-microglobulin and CD298, and then washed. The cells from both conditions were then pooled and stained with a panel of oligo-conjugated antibodies against surface proteins, followed by washing. Next the cells were counted and loaded into one well of a Chromium Next GEM Chip K (10X Genomics, P/N 2000182), and run on a 10X Chromium Controller. Gene expression, V(D)J, and cell surface protein libraries were constructed as per the manufacturer’s protocol using Chromium Next GEM single Cell 5’ Reagent Kits v2 (Dual Index). The run was sequenced on a NovaSeq SP flow cell.

Second scRNA-sequencing run (Th1-like, CD30): Th1-like cells were generated as described in the previous sections (starting condition: for each donor, 8x10^5^ freshly isolated naive CD4^+^ T cells were resuspended in 10 mL base media supplemented with Th1 cocktail and CD3/CD28 beads). The differentiation period resulted in 23.4x10^6^ cells for donor 1, 21.75x10^6^ cells for donor 2, 14.46x10^6^ for donor 3, and 19.05x10^6^ cells for donor 4. The cells were then concentrated into 1 mL volume each in 2% FBS 1% P/S 2 mM EDTA PBS, and 10 µL anti-CD30-Biotin was added to each sample (BD Biosciences, P/N 555828). After 20 minutes of incubation with the biotinylated antibody, the cells were washed twice and then the manufacturer’s protocol was followed to obtain magnetic particle free CD30 enriched and CD30 depleted fractions of cells (STEMCELL Technologies, P/N 17653). This process resulted in 13.2x10^6^ depleted cells and 3.72x10^6^ enriched cells for donor 1, 10.5x10^6^ depleted and 2.9x10^6^ enriched cells for donor 2, 8.43x10^6^ depleted and 2.81x10^6^ enriched cells for donor 3, 11.1x10^6^ depleted and 3.63x10^6^ enriched cells for donor 4. 1x10^6^ cells from each of these fractions were plated in 1 mL base media with and without CD3/CD28 beads at a ratio of 1 bead per 8 cells for 24 hours. After 24 hours, the beads were removed using a DynaMag-2 Magnet (Invitrogen, P/N 12321D), cells were counted, and 2x10^5^ cells from each donor were combined into pools for each condition: CD30^-^, CD30^-^ stimulated, CD30^+^, CD30^+^ stimulated. Cells from each condition were strained through 40 µm cell strainers, counted, and then 5.5x10^4^ from each condition loaded into 4 individual lanes of a Chromium Next GEM Chip K. The gene expression library for this run was constructed as per the manufacturer’s protocol using Chromium Next GEM single Cell 5’ Reagent Kits v2 (Dual Index). The run was sequenced on a NovaSeq S2 flow cell.

### 2.11 Enrichment of single-cell HGF transcripts

HGF transcripts were amplified from 50 ng of cDNA using a two-step nested PCR inspired by the genotyping of transcripts protocol (31). The first PCR used the generic sample indexing forward primer for 10X Chromium (SI-PCR: AATGATACGGCGACCACCGAGATCTACACTCTTTCCCTACACGACGC*T*C) and an outer reverse primer targeting the HGF transcript containing (PCR1_HGF: AGCAAGTGAGAAGCATCGTGTCGTATTTCTTCTTTTCCTTTGTCCCTCTGC) and was amplified for 14 cycles of 98°C for 20 s, 58°C for 30 s and 72°C for 20 s. Products were cleaned up and size-selected by SPRI bead selection (0.45× supernatant, 0.6× supernatant, and 1.4× bead fraction). The second PCR used the SI-PCR forward primer and a nested reverse primer targeting the previous HGF amplicon as well as an overhang containing the PCR handle for the Illumina TruSeq Small RPI-x primer used for indexing (PCR2_HGF: CACCCGAGAATTCCAGCTGGCAGGAGTTTGGTCAC*C) and was amplified for 16 cycles of 98°C for 20 s, 58°C for 30 s and 72°C for 20 s. Products were cleaned up and size-selected by double-sided (0.7× and 1.6×) SPRI bead selection. Amplicons were quantified using the Qubit4 with dsDNA high-sensitivity kits. A finished sequencing library was generated by an indexing PCR using SI-PCR forward primer and an RPI-x reverse-indexing primer (RPI-x: CAAGCAGAAGACGGCATACGAGATXXXXXXXXXXGTGACTGGAGTTCCTTGGCACCCGAGAATTCCA) for 7 cycles of 98°C for 20 s, 54°C for 30 s and 72°C for 20 s and followed by a final 1.5X SPRI bead selection.

### 2.12 Single-cell RNA-sequencing data

Gene expression and TCRαβ libraries were processed using Cell Ranger (v6.0.1; 10X Genomics). HGF-enriched libraries were quantified using Kallisto-Bustools (32). For the Th1 run, hashtags and surface antibody libraries were quantified with Kallisto-Bustools using the KITE workflow. For Th1 run, samples were demultiplexed using Seurat::HTODemux (positive.quantile= 0.99). For CD30 enriched run, samples were demultiplexed by genotype using vireo (33). The datasets were analyzed using R and the Seurat 4.3.0 package (34, 35). Low quality cells were filtered based on having less than 800 or 1000 detected genes or more than 30% or 20% of their total unique molecular identifiers (UMIs) stemming from mitochondrial transcripts for the Th1 and CD30-enriched runs, respectively. For the Th1 run, Cell cycle scoring was done using Seurat:: CellCycleScoring and mRNA counts were normalized using Seurat::SCTransform regressing out S- and G2M scores to reduce clustering by cell cycle status. Cells were clustered and visualized based on both mRNA and Surface protein expression using Seurat:: FindMultiModalNeighbors (WNN) using the first 20 and 13 principal components, respectively. For the CD30 enriched run, after selecting the top 10% most variable genes and principal component analysis, the first 24 principal components from were used for clustering and UMAP visualization. Additional R packages used: ggplot2, patchwork, dplyr, and pheatmap (36–39).

### 2.13 Flow cytometry

For surface staining, antibodies were diluted in brilliant stain buffer (BD Biosciences, P/N 563794) and cells were stained with this mixture. Washing steps were performed with a standard flow cytometry buffer (1% FBS, 0.02% NaN_3_, PBS). For intracellular staining, the Foxp3/Transcription Factor Staining Buffer Set (Invitrogen, P/N 00-5523-00) was used according to the manufacturer’s instructions. Cells were pretreated with GolgiStop for cytokine staining (BD, P/N 554724). Viability was assessed using either 7-AAD (BioLegend, P/N 420403) or fixable viability dye eFluor780 (Invitrogen, P/N 65-0865-14). All antibodies used were from BD Biosciences: CD3-PE (P/N 566683), CD4-FITC (P/N 300538), CD4-BV786 (P/N 563877), IFN-γ-FITC (P/N 554551), Isotype control-FITC (P/N 554679), CD9-PE (P/N 555372), CD30-BV421 (P/N 562876), CD45RA-BV711 (P/N 612847), CD45RO-BV421 (P/N 562641), CD52-AF647 (P/N 563610), CD96-BV711 (P/N 563174).

### 2.14 Fluorescence-activated cell sorting

A BD FACS ARIA-II was used to sort PSO-2 cells. 1x10^7^ normal PSO-2 cells from a culture flask were stained in 1 mL 10% HS PBS containing antibodies against CD9, CD30, CD52, and CD96. After washing steps, 7-AAD was added to the cell suspension and then the cells were sorted into CD96^-^CD52^-^CD9^+^CD30^low^ (13.4% of the total cells) and CD96^-^CD52^-^CD9^-^CD30^+^ (6.72% of the total cells) fractions under sterile conditions, at room temperature, into PSO-2 cell culture media with 20% HS (see cell culture section). Small debris and doublets were gated out, and dead cells were excluded by 7-AAD staining. The sort resulted in 3.3x10^5^ CD96^-^CD52^-^CD9^-^CD30^+^ cells with a viability of 95.7% and 5.48x10^5^ CD96^-^CD52^-^CD9^+^CD30^low^ cells with a viability of 90.3%. 3.26x10^5^ cells from each fraction, as well as an unsorted control sample were cultured in 1 mL PSO-2 media for 24 hours in a 24 well plate. After 24 hours, supernatant and cell pellets were collected, and this material was tested for HGF protein by HGF ELISA and HGF gene expression by qPCR.

### 2.15 Statistical analysis

GraphPad Prism 9.5.1 (GraphPad Software, San Diego, California USA, www.graphpad.com) was used for statistical analysis. The type of test used is indicated in the figure legends.

## 3 Results

### 3.1 Memory CD4^+^ T cells from the peripheral blood of healthy human donors are HGF positive

**Figure 1:**
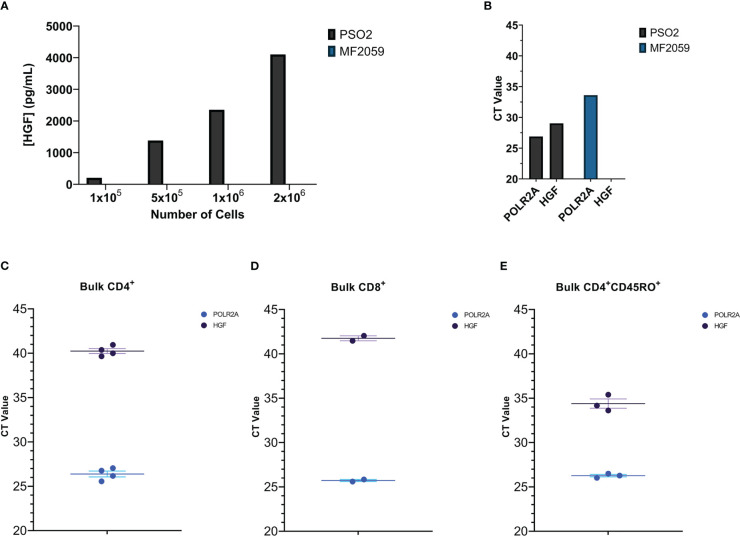
**(A)** Several concentrations of PSO-2 and MF2059 cells were cultured in 1 mL media for 24 hours. The supernatants were tested for HGF protein by HGF ELISA. MF2059 produced no detectable HGF protein. **(B)** Material from PSO-2 and MF2059 was tested for HGF mRNA by qPCR. POLR2A is a reference gene (40). While MF2059 do have expression of the reference gene, there was no detectable HGF. **(C–E)** qPCR of **(C)** Bulk CD4^+^ T cells, **(D)** Bulk CD8^+^ T cells, or **(E)** Bulk CD4^+^CD45RO^+^ memory T cells that were purified from buffy coats and then immediately pelleted and frozen.

The human CD8^+^ T cell line PSO-2 spontaneously produces large amounts of HGF protein and mRNA (**Figures 1A, B** respectively, **Supplemental Figures 1A, B**; MF2059 is a HGF^-^ T cell line used as a negative control). We hypothesized that human primary T cells can also produce HGF. Freshly isolated bulk CD4^+^, CD8^+^, and CD4^+^CD45RO^+^ T cells were tested for HGF mRNA, and memory CD4^+^ T cells were found to have relatively high basal HGF gene expression, indicating that activated CD4^+^ T cells could be HGF positive (**Figures 1C–E**, respectively).

### 3.2 Human naive CD4^+^ T cells that have been differentiated into Th1-like cells are positive for HGF protein and mRNA

**Figure 2:**
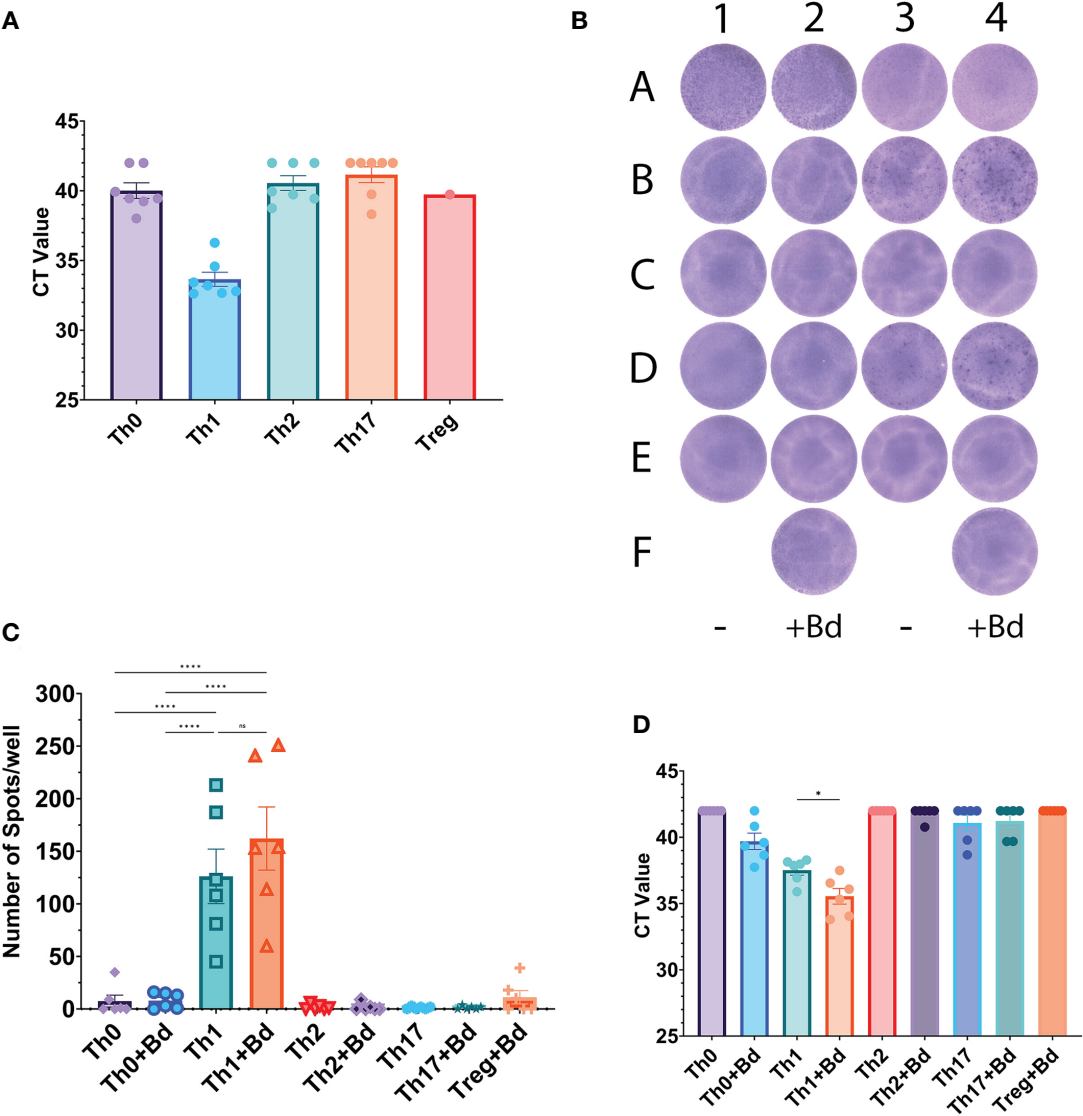
**(A)** Pre-ELISpot samples. Prior to loading into the ELISpot plate, samples from the same batch of cells were taken and tested by qPCR for HGF mRNA. CT values of 42 were manually entered if HGF was not detected. Although all of the Th1 samples are positive for HGF mRNA, statistical tests were not performed on this data since several samples in the Th0, Th2, and Th17 groups were negative for HGF mRNA. Treg-like cells only have one data point due to low number of cells, and the ELISpot was the priority. Note: data from an extra donor (donor 7) is included here, but cells from this donor were not used for the subsequent ELISpots. **(B)** HGF ELISpot. The wells of columns 2 and 4 have been treated with beads coated in anti-CD3/CD28 antibodies at a ratio of 1 bead per 8 cells (+Bd). Cell lines: A1-2 PSO-2, A3-4 MF2059 (see **Supplemental Figure 1** for more information on these cell lines). The rest of the wells contain cells that have been *in vitro* differentiated into Th-like subsets. Donor 1: B1-2 Th0-like, B3-4 Th1-like, C1-2 Th2-like, C3-4 Th17-like, F2 Treg-like. Donor 2: D1-2 Th0-like, D3-4 Th1-like, E1-2 Th2-like, E3-4 Th17-like, F4 Treg-like. **(C)** Summary data from three separate ELISpot plates representing 6 donors. See **Supplemental Table 2** for multiple comparison data between all of the groups. **(D)** Post-ELISpot samples; after 24-hour incubation in the ELISpot plate, the cells were recovered and then processed for qPCR. The graph shows CT values for HGF expression of all 6 donors that were tested. A CT value of 42 was manually entered if HGF was not detected. All of the groups contained samples that were negative for HGF, except for Th1 and Th1+Bd, so statistical tests comparing Th1 to other groups were not conducted. Data in graphs is presented as mean ± SEM. Statistics shown in graphs **(C)** and **(D)** are the result of a one-way ANOVA with Tukey’s multiple comparisons test. P values: ns (P > 0.05), * (P ≤ 0.05), **** (P ≤ 0.0001).

*In vitro* differentiation of naive CD4^+^ T cells into Th-like subsets was used to assess differences in HGF expression between the subsets. After five days of differentiation into Th-like cells, the cells that had been treated with a Th1 cocktail containing IL-12 were the most positive for HGF mRNA (**Figure 2A**). The Th-like cells were then plated into an HGF ELISpot for 24 hours, and the Th1-like cells produced the most spots (**Figures 2B, C**). After the ELISpot incubation period, cells were recovered from the ELISpot plate and tested by qPCR, and the Th1-like cells were again the most positive for HGF mRNA (**Figure 2D**). Importantly, stimulation of Th1-like cells with anti-CD3/CD28 beads increased the number of spots produced in the HGF ELISpot, and the amount of HGF mRNA detected by qPCR, indicating that TCR-mediated signaling induces expression of HGF (**Figures 2C, D**).

### 3.3 Human naive CD4^+^ T cells become positive for HGF mRNA by day 3 of *in vitro* differentiation into Th1-like cells

**Figure 3:**
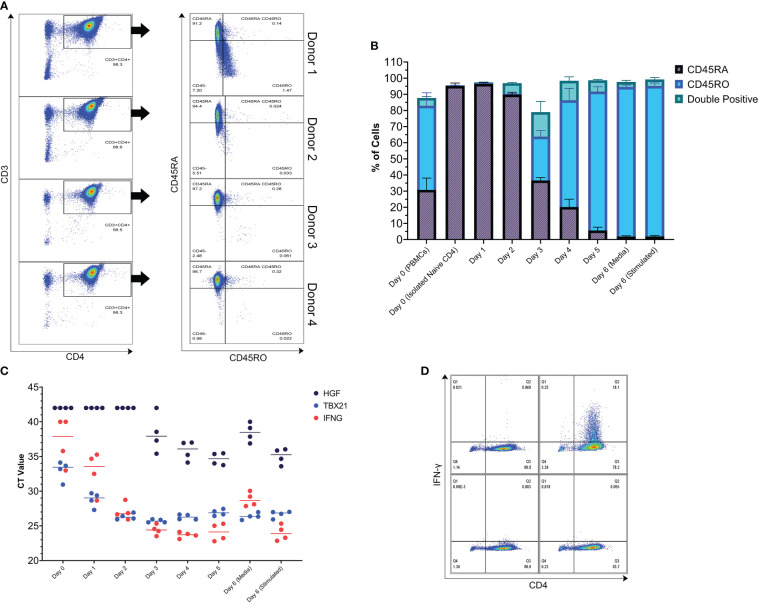
Naive CD4^+^ T cells were isolated from 4 donors and used to conduct a kinetics study. **(A)** Purity of the isolated Naive CD4^+^ T cells assessed by flow cytometry. Left column: dot plots with CD3 on the y-axis and CD4 on the x-axis. Right column: dot plots of the CD3^+^CD4^+^ population with CD45RA on the y-axis and CD45RO on the x-axis. 98.43% of the cells were CD3^+^CD4^+^ and 95.13% of those cells were CD45RA^+^ (average of the 4 donors). Small debris, doublets, and dead cells have been gated out prior to the CD3^+^CD4^+^ gate. **(B)** Kinetics of CD45RO and CD45RA surface protein expression of the isolated naive CD4^+^ T cells as they undergo *in vitro* differentiation towards a Th1-like phenotype. The graph is a visualization of data that was collected by flow cytometry. After 5 days of differentiation, the anti-CD3/CD28 beads were removed and the cells were put into fresh media without differentiation cytokines and then left in this media or restimulated with anti-CD3/CD28 beads (ratio 1 bead to 8 cells) for 24 hours, representing the typical experimental period used in this study. **(C)** Kinetics of HGF and Th1 gene expression over the typical experimental period. **(D)** IFN-γ protein production assessed by flow cytometry. Left column: unstimulated cells. Right column anti-CD3/CD28 bead stimulated cells (ratio 1 bead to 8 cells). Cells were treated with monensin during the last 4 hours of the 24-hour stimulation period. Top panels: cells stained intracellularly with anti-IFN-γ-FITC antibody. Bottom panels: cells stained intracellularly with isotype-FITC antibody.

Isolation of T cells from the blood of 4 healthy donors yielded highly pure populations of naive CD4^+^ T cells (**Figure 3A**). During *in vitro* differentiation in Th1 media, these cells were initially 95.13% CD45RA^+^ (average of the 4 donors), and by day 3 there was a shift in surface CD45 isoform expression to CD45RO, CD45RA, and double positive populations. By day 4, most of the cells were CD45RO positive, indicating mass activation of the population over the differentiation period (**Figure 3B**, see gating strategy in **Supplemental Figure 2A**). Looking at gene expression, expression of the characteristic Th1 genes TBX21 and IFNG increased by day 1, with maximum expression reached by day 3 (**Figure 3C**). The expression levels of IFNG and TBX21 were high, in agreement with the T-bet master transcription factor model and characteristic cytokine for the Th1 phenotype (41). In addition, cells differentiated and stimulated using the same process were positive for IFN-γ protein (**Figure 3D**). In comparison to Th1-like cells, Th2-like cells had low expression of IFNG despite stimulation with anti-CD3 and anti-CD28 coated beads for 24 hours (**Supplemental Figure 2C**). In addition, Th2-like cells had lower expression of TBX21, the Th1 transcription factor, and higher expression of GATA3 and IL5, genes associated with Th2 cells (**Supplemental Figure 2C**) (28). Importantly, HGF gene expression by bulk memory CD4^+^ T cells sampled immediately after purification from PBMCs was similar to Th1-like cells post 5-day differentiation period (**Figures 1E** and **3C**; memory CD4^+^ T cells treated with Th1 cocktail kinetics in **Supplemental Figure 2B**).

### 3.4 Single-cell RNA-sequencing of Th1-like cells revealed CD30 as a promising surface marker of HGF positive cells

**Figure 4:**
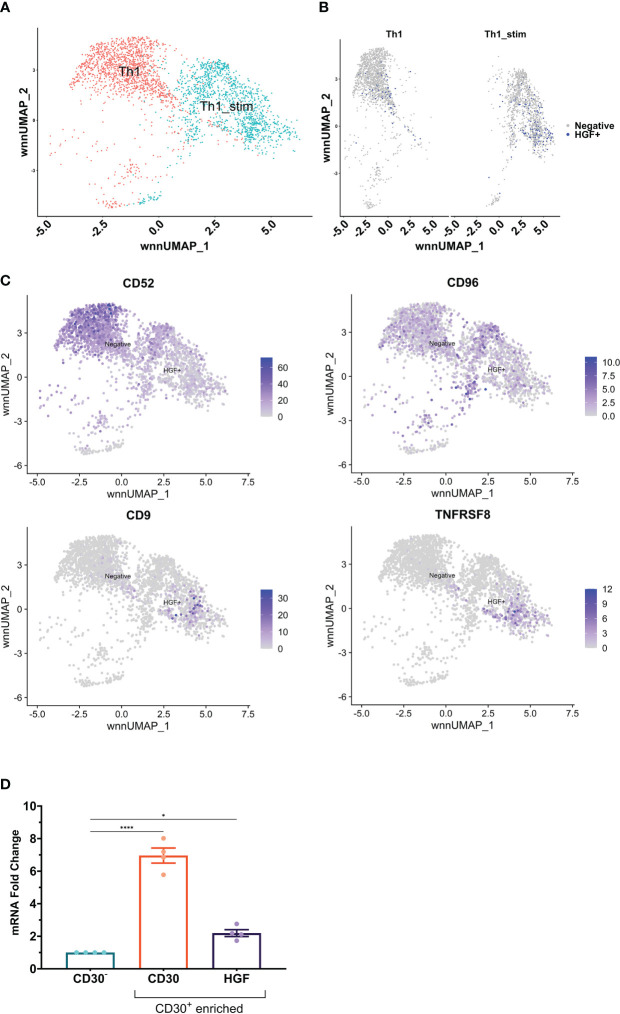
**(A)** scRNA-seq was performed on Th1-like cells from one donor that had been split into two samples; stimulated with anti-CD3/CD28 beads (ratio 1 bead to 8 cells) for 24 hours, or unstimulated. In the UMAP, the two samples cluster slightly apart from each other, indicating difference between the stimulated and unstimulated samples. **(B)** Split view of the samples with cells that had ≥ 2 HGF transcripts highlighted in blue. **(C)** Differential gene expression analysis as well as manual screening indicated several markers which could be useful for enrichment of HGF^+^ cells and/or depletion of HGF^-^ cells. CD52 and CD96 are mainly expressed in HGF^-^ cells, while CD9 and TNFRSF8 (CD30) are mainly expressed by HGF^+^ cells. **(D)** Naive CD4^+^ T cells from 4 donors were differentiated into Th1-like cells. CD30^+^ cells were enriched from the Th1-like pools using a biotinylated anti-CD30 antibody and a biotin positive selection kit. The resultant CD30 enriched samples were compared to the remaining (CD30 depleted) cells by qPCR for CD30 and HGF genes. Statistics in **(D)** are the result of a one-way ANOVA with Dunnett’s multiple comparisons test. P values: ns (P > 0.05), * (P ≤ 0.05), **** (P ≤ 0.0001).

In the HGF ELISpot data, only 0.03% of the cells that were put into the well of the stimulated Th1 treatment group released HGF protein. We hypothesized that it would be possible to identify a surface marker which would enable isolation of a more HGF positive subset of CD4^+^ T cells. Because there are no suitable HGF flow antibodies, we utilized single-cell RNA-sequencing (scRNA-seq) of Th1-like cells. Targeted amplification of HGF transcripts enabled identification of HGF positive cells in the sequencing library, and there were more positive cells in the stimulated group compared to the unstimulated group, in agreement with previous results (**Figures 4A, B**). T cell receptor sequence diversity was normal in HGF^+^ and HGF^-^ cells, with no difference in clonality between the groups (data not shown). Differential gene expression analysis of the data, as well as manual screening based on a custom list of known genes that encode surface proteins revealed several markers which might be suitable for enrichment of HGF positive cells and/or depletion of negative cells. Among this list, CD52, CD96, CD9, and TNFRSF8 (CD30) were the most promising because these markers were expressed mainly by the HGF^+^ or HGF^-^ populations, but not both (**Figure 4C**). PSO-2 cells were sorted by fluorescence-activated cell sorting into CD96^-^CD52^-^CD9^+^CD30^low^ and CD96^-^CD52^-^CD9^-^CD30^+^ fractions, and these cells were compared to an unsorted PSO-2 population by ELISA and qPCR testing for HGF. Though there was no difference in the ELISA results, there was slightly higher HGF gene expression in the CD96^-^CD52^-^CD9^-^CD30^+^ fraction (see **Supplemental Figures 3A, B**). Because PSO-2 already have high expression of HGF, even a slight increase indicated enrichment. Furthermore, > 95% of the typical unsorted PSO-2 population is positive for CD30 (see **Supplemental Figure 3C**). Meanwhile, Th1-like cells were found to express surface CD30 protein, and this level increased slightly with CD3/CD28 stimulation (**Supplemental Figure 3D**). Human naive CD4^+^ T cells from 4 donors differentiated into Th1-like cells, and then processed using an anti-CD30-Biotinylated antibody and a biotin positive selection kit yielded CD30 depleted (CD30^-^) and enriched (CD30^+^) populations. Tested by qPCR, the CD30^+^ population had 6.96-fold more CD30 mRNA and 2.2-fold more HGF mRNA compared to the CD30^-^ population, representing significant differences (**Figure 4D**).

### 3.5 CD30^+^ enriched Th1-like cell populations have a higher proportion of cells positive for HGF transcripts

**Figure 5:**
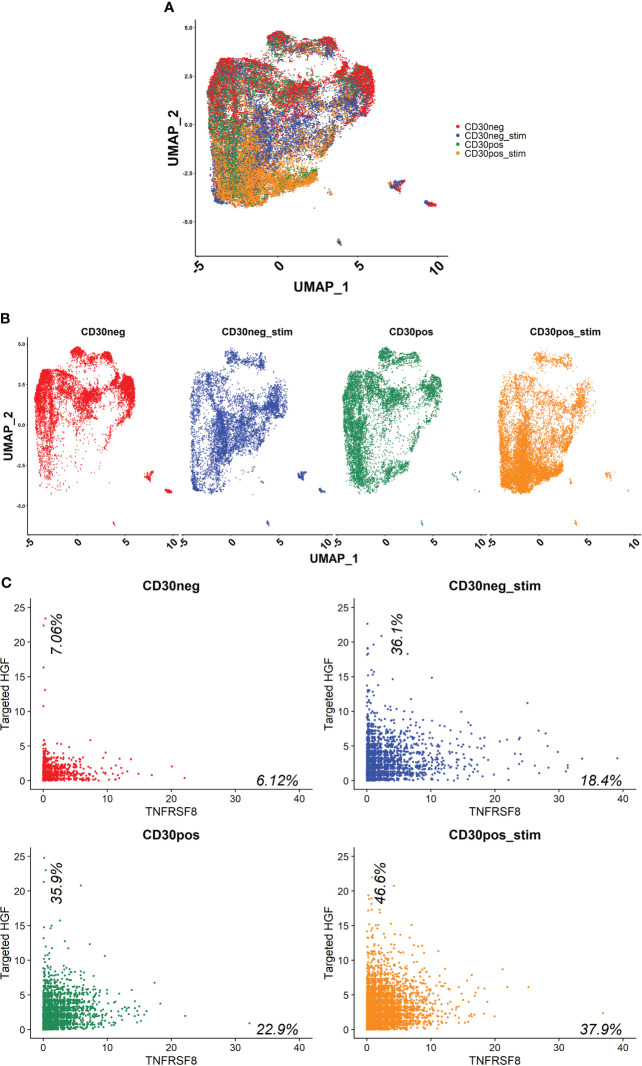
scRNA-seq was performed on Th1-like cells from four healthy donors that had been split into four samples per donor. First, the cells were processed into CD30 enriched and CD30 depleted fractions, and then these fractions were further split into stimulated or unstimulated samples. The cells from each condition were pooled together, resulting in 4 samples which each contained the cells from 4 donors; CD30^-^, CD30^-^ stimulated, CD30^+^, and CD30^+^ stimulated (stimulated = stimulated with anti-CD3/CD28 beads for 24 hours). **(A)** UMAP showing how the samples clustered. **(B)** UMAP of individual samples. **(C)** Scatter plots of each sample where HGF transcripts are on the y-axis and TNFRSF8 (CD30) transcripts are on the x-axis. The data shown are raw counts of transcripts. Percentages on the graphs indicate what proportion of the cells had ≥2 HGF transcripts on the y-axis and ≥1 CD30 transcript on the x-axis. HGF transcripts were detected using a targeted amplification method (see materials and methods).

**Figure 6:**
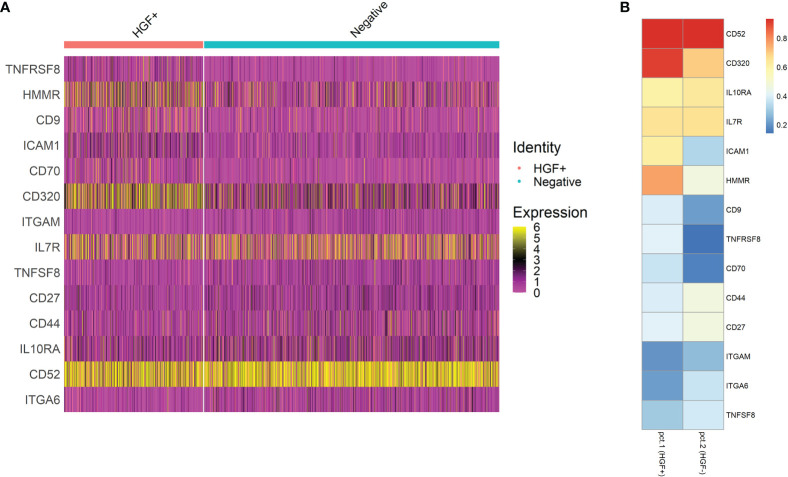
Differential gene expression testing was performed by comparing HGF^+^ cells to HGF^-^ cells and by filtering out genes that were not expressed by at least 25% of the cells in either group. The genes that were tested were based on a list of 391 genes that encode surface proteins. The resulting genes of interest are shown as a feature expression heatmap in **(A)** And a heatmap with easier to read percentage positive values visualized in **(B)**. See **Supplemental Figure 4** for a more extensive heatmap of differentially expressed genes which was not limited to pre-selected genes.

Human naive CD4^+^ T cells from four donors were differentiated into Th1-like cells over 5 days, and then processed into CD30^+^ and CD30^-^ populations. These populations were then split into stimulated and unstimulated groups, and after 24 hours scRNA-seq was performed. The treatment groups clustered closely together, indicating no large differences in the populations (**Figure 5A**). However, there were differences in the clusters that could be observed when the samples were split into individual groups, such as more cells clustering in the central and lower regions in the stimulated groups (**Figure 5B**). The CD30^-^ group had the lowest HGF expression where 7.06% of the cells had ≥ 2 HGF transcripts (HGF^+^). When CD30^-^ cells were stimulated, this number increased to 36.1%. Meanwhile, in the unstimulated CD30^+^ group 35.9% were positive, and the stimulated CD30^+^ group had the highest proportion of positive cells at 46.6% (**Figure 5C**). Differential gene expression analysis based on a custom list of known genes that encode surface proteins revealed an updated list of surface markers that are differentially expressed in HGF^+^ versus HGF^-^ cell populations (**Figures 6A, B**). In agreement with the results of the first scRNA-seq run, CD9 and CD30 appear as upregulated HGF^+^ markers in this list. Interesting new prospects include the highly expressed genes HMMR and CD320. Aside from the surface markers, there are many genes with differential expression between the HGF^+^ and HGF^-^ groups (top 200 upregulated and all downregulated genes that were expressed in at least 25% of the cells are shown in **Supplemental Figures 5A, B**, respectively). We hypothesized that sCD30L, the ligand for CD30, may affect HGF expression in Th1-like cells. However, we found no difference between treated and untreated cells (data not shown).

### 3.6 Exogenous HGF protein does not affect TNF or MRC1 gene expression in human peripheral blood monocyte‐derived macrophages

**Figure 7:**
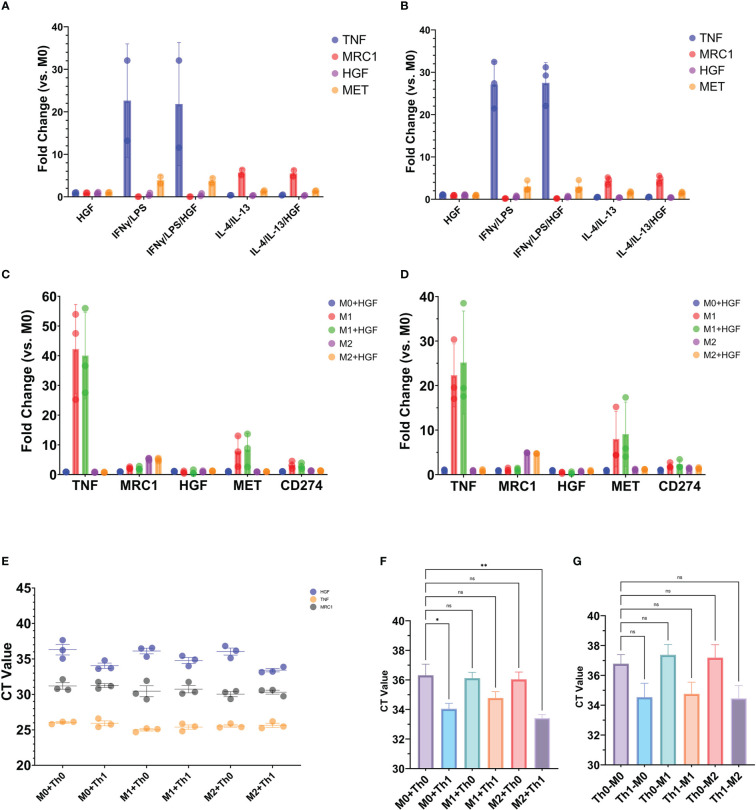
Exogenous HGF protein **(A–D)** or donor matched T cells **(F, G)** added to macrophage cell cultures. Monocytes purified from the blood of healthy human donors were differentiated into macrophages for 5 days in M-CSF media **(A, C, E, F)** or GM-CSF media **(B, D)**. Macrophages were treated with or without 50 ng/mL HGF protein during 24-hour treatment with **(A)** an M1 cocktail containing IFN-γ and LPS or **(B)** an M2 cocktail containing IL-4 and IL-13. **(C, D)** Macrophages were treated with or without 50 ng/mL HGF protein for 24 hours after polarization into M1 or M2. The graphs show qPCR data of gene expression of TNF and MRC1 to indicate M1 or M2 phenotype, respectively. **(E–G)** Macrophages that had been differentiated into M1, M2, or unstimulated M0 were co-cultured with donor matched Th0 or Th1-like CD4^+^ T cells and CD3/CD28 beads for 24 hours. After the 24-hour period, T cells and beads were resuspended in the wells and processed for testing. The well was then washed, and the remaining contents processed for testing. **(E, F)** qPCR data generated from RNA extracted from the cells that were adherent to the wells after removal of T cells and washing. **(G)** qPCR data generated from RNA extracted from T cells that were removed from the wells. Statistics in **(F, G)** are the result of a one-way ANOVA with Dunnett’s multiple comparisons test. P values: ns (P > 0.05), * (P ≤ 0.05), ** (P ≤ 0.01).

Recently it was shown that HGF can shift the polarization of mouse bone marrow derived macrophages from the inflammatory M1 phenotype towards the anti-inflammatory M2 (42). It could be possible that Th1-like cells producing HGF can affect the phenotype of macrophages, therefore we sought to reproduce these results with human cells by isolating monocytes from the blood of healthy donors, differentiating the monocytes into macrophages, and then polarizing the macrophages into the M1 or M2 phenotype. However, when exogenous HGF was administered during M1 or M2 differentiation, there was no effect on genes associated with the respective phenotypes (**Figures 7A, B**). HGF protein added for 24 hours after the macrophages had been differentiated into M1 or M2 phenotype also had no effect on the expression of these genes (**Figures 7C, D**). Despite these results, we proceeded to conduct a donor matched co-culture experiment where differentiated macrophages were incubated with either Th0 or Th1-like CD4^+^ T cells. The type of Th-like cell added to the wells did not have an appreciable effect on TNF or MRC1 gene expression. On the other hand, HGF expression was significantly greater when M0 or M2 macrophages had been in co-culture with Th1-like cells, with the highest expression detected when Th1-like and M2 macrophages had been in culture together (**Figures 7E–G**).

### 3.7 Expression of HGF can be pharmacologically manipulated with existing drugs

**Figure 8:**
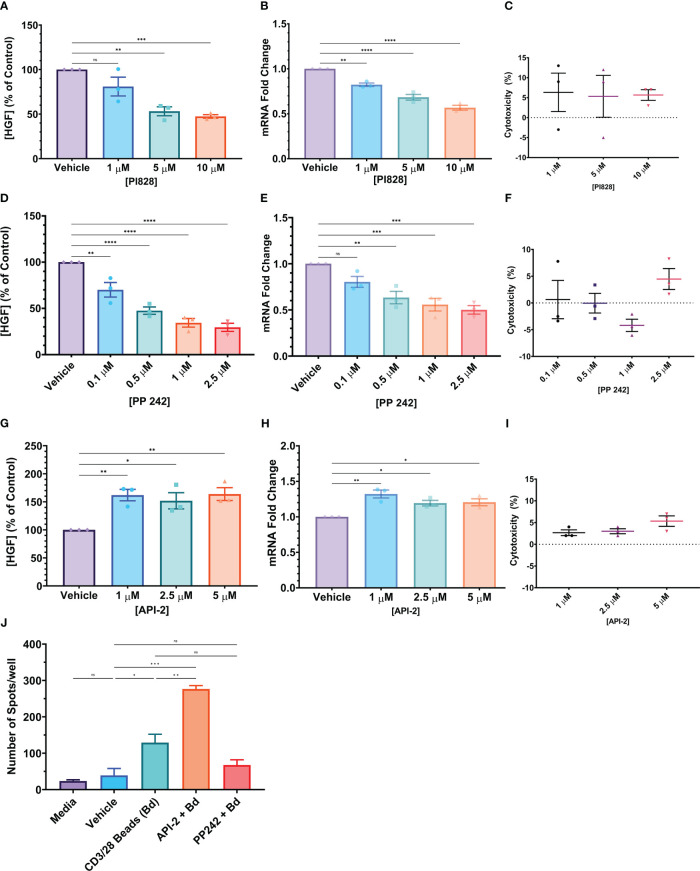
**(A–I)** Small molecule kinase inhibitors (SMKI) tested on the PSO-2 cell line. In all cases, 5x10^5^ PSO-2 cells were incubated in 1 mL media with the listed concentration of SMKI or a vehicle control for 24 hours. After this period, the supernatant or cells were tested for HGF with either an HGF ELISA or qPCR. **(C, F, I)** The supernatants were also screened for toxicity by LDH assay. **(A–C)** PI3K inhibitor PI828. **(D–F)** mTORC1/C2 inhibitor PP242. **(G–I)** Akt inhibitor API-2 (Triciribine). **(J)** 1x10^6^ post-differentiation Th1-like cells per well were incubated in an HGF ELISpot in media, vehicle (DMSO), with anti-CD3/CD28 beads (ratio 3 beads to 5 cells, Bd), beads in combination with the Akt inhibitor API-2 (API-2 + Bd), or beads in combination with the mTORC1/C2 inhibitor PP242 (PP242 + Bd) for 24 hours. The resultant spots were counted and are presented as number of spots per well in the graph. Data in graphs is presented as mean ± SEM. Statistics shown in graphs **(A, B, D, E, G, H)** were calculated with one-way ANOVA with Dunnett’s multiple comparisons test. Statistics in **(J)** were calculated with a one-way ANOVA with Tukey’s multiple comparisons test. P values: ns (P > 0.05), * (P ≤ 0.05), ** (P ≤ 0.01), *** (P ≤ 0.001), **** (P ≤ 0.0001).

The PSO-2 cell line produces HGF spontaneously. When PSO-2 was treated with ionomycin and PMA, mimicking T cell receptor signaling, HGF levels increased (**Supplemental Figures 5A, B**)(43, 44). We hypothesized that HGF signal transduction must involve kinases downstream of the TCR signal transduction pathway, and tested inhibitors of known TCR-mediated signaling pathways including NFAT, CREB, NF-κB, PI3K, and MEK. Of these, only the phosphoinositide 3-kinase (PI3K) inhibitor PI828 resulted in significant difference in HGF protein and mRNA levels. Therefore, PI3K was targeted using PI828 at sub-toxic concentrations (<10% average toxicity in 24 hours, assessed by LDH) (45). PI828 significantly inhibited HGF at both the protein and mRNA levels, reducing HGF protein produced to 47.35% of the control, and 1.77-fold reduction of mRNA (**Figures 8A–C**). Further downstream, greater inhibition of HGF was achieved by targeting mTORC1/C2 with PP242 (46). At the protein level, this inhibitor reduced HGF production to 29.55% of control, and at the mRNA level, 2-fold reduction (**Figures 8B–D**). Because the mTORC1 inhibitor rapamycin did not have as strong of an effect as the dual mTORC1/C2 inhibitor PP242 (**Figures 2E, F** and **Supplemental Figures 5C, D**), we hypothesized that mTORC2 could be important in the HGF signal transduction pathway. mTORC2 phosphorylation of Akt S473 is necessary for full activation of Akt (47). Therefore, we looked upstream of mTORC1 and tried inhibiting Akt with API-2, also known as triciribine. We expected that inhibiting Akt would decrease HGF, however API-2 increased HGF at both the protein and mRNA levels, to 163.79% of control and 1.21-fold change, respectively (**Figures 8G–I**). To confirm that these cell line results translate to human primary CD4^+^ T cells, Th1-like cells were generated and then plated into an HGF ELISpot (**Figure 8J**). Th1-like cells that were treated with anti-CD3/CD28 coated beads and API-2 produced significantly more spots than cells that were only treated with the stimulatory beads. On the other hand, although not significantly different, the mean number of spots was lower in the group that was treated with both PP242 and anti-CD3/CD28 coated beads compared to the group that was stimulated with beads alone. Human naive CD8^+^ T cells that had undergone the same differentiation process used to generate Th1-like cells were treated in the same way, and yielded similar results, although the CD8^+^ cells produced more spots in general than their CD4^+^ counterparts (**Supplemental Figure 5E**).

## 4 Discussion

The observation that human CD4^+^ T cells produce HGF protein has been reported previously; however, the observation was made regarding a T cell line and study was not continued (23). Interestingly, we also observed that a human CD8^+^ T cell line, PSO-2, spontaneously produced HGF protein, and this inspired further study into the topic. To our knowledge, our data represents the first evidence that human CD4^+^ T cells purified from the blood of healthy donors are positive for both HGF mRNA as well as protein. In addition, we also show preliminary data indicating that the same is true for CD8^+^ T cells.

In this study, we have shown that memory CD4^+^ T cells from the peripheral blood of healthy donors are positive for HGF mRNA, and that *in vitro* differentiated Th1-like cells are positive for HGF mRNA and protein. Initially, we observed an interesting pattern when testing T cell supernatant in an HGF ELISA, however the values detected were well below the detection limit of the assay. This observation motivated us to seek more sensitive methods, and as a result we developed an HGF ELISpot. The HGF ELISpot enabled comparison of *in vitro* differentiated CD4^+^ T cell subsets, and the data obtained revealed that Th1-like cells were the most positive. From there we sought to use flow cytometry to identify surface markers that might enable enrichment of HGF producing cells. However, numerous attempts to detect surface bound or intracellular HGF protein using several anti-HGF antibody clones, host species, staining methods, and systems all failed. We thought that perhaps markers could be discovered through scRNA-seq, however, in our experience this method was not sensitive enough to detect cytokines with low expression levels. Therefore, we employed a nested amplification method to specifically target and amplify HGF mRNA transcripts, which enabled economical detection of HGF positive cells. With this method we were able to identify and show that CD30 can be used to enrich HGF positive cells and found additional markers which could prove useful for the purpose of further enrichment.

The *in vitro* differentiation method we used gave an idea of what a given type of T cell will express in the controlled environment of abundant antigen, co-stimulation, specific exogenous cytokine and chemical milieu, and neutralization of specific cytokines that the cells may release and potentially react to. Much like using ionomycin and PMA to see which cytokines cells produce, this method may represent an extreme snapshot of the cells in highly polarized states which may not exist naturally, and the purpose of using the method was to get an idea of what could be happening *in vivo* (48, 49). For that reason, we designated our *in vitro* differentiated cells as “Subset-like” (e.g., Th1-like). Although TBX21 has been described as the so-called master transcription factor for Th1 cells, expression of TBX21 by several types of immune cells has been described including natural killer cells, B cells, and dendritic cells (41, 50). Thus, it was important that we verified the purity of our isolated naive CD4^+^ T cell populations (**Figures 3A, B**) and confirmed that Th1-like cells are positive for IFN-γ protein (**Figure 3D**). It was an interesting finding that freshly isolated bulk CD4^+^ memory T cells had relatively high basal HGF mRNA expression that was comparable to *in vitro* differentiated Th1-like cells at days 5 and 6 (**Figures 1E** and **3C**, respectively). This supports the idea that HGF^+^ CD4^+^ T cells may exist naturally *in vivo*. However, this data must be interpreted with caution because the enriched CD45RO^+^ memory T cell populations could have been contaminated with other HGF^+^ cell populations. When isolated memory CD4^+^ T cells were treated with the Th1 differentiation cocktail, the mean HGF expression level decreased over the treatment period (**Supplemental Figure 2B**). Rivino et al. have shown that circulating memory T cells are more positive for IFN-γ than IL-4, especially when stimulated, but also that this population contains cells poised to polarize into either Th1 or Th2 when reactivated (51). Our *in vitro* testing of differentiated naive CD4^+^ T cells shows that Th1-like cells are positive for HGF, but Th2-like are not. If the memory T cells tested contained discrete populations that polarized into Th1 and Th2 memory CD4^+^ T cells when restimulated, then this could explain the trajectory of the HGF mRNA expression level during the experimental period. In the context of HGF expression, it would be interesting to further study CD4^+^ memory T cells. CD4^+^CCR7^-^CXCR3^+^ effector memory T cells could be especially interesting as this cell type has been shown to produce large amounts of IFN-γ protein upon restimulation, possibly representing a pool of memory Th1 cells that were generated *in vivo* (51).

CD30 has been shown to be an inducible co-stimulatory molecule on mouse CD4^+^ T cells, and ligation of CD30 was shown to increase proliferation (52). The co-stimulatory effect of CD30 ligation on cell proliferation was more effective than CD28 co-stimulation, but this was only the case when the cells were stimulated with low concentrations of anti-CD3 antibody (52). In our study, CD30 gene expression was inducible in a CD30 depleted CD4^+^ T cell population upon anti-CD3/CD28 stimulation (**Figure 5C**). However, treatment of Th1-like cells with sCD30L did not affect HGF expression. Thus, CD30 was considered to be a tool for enrichment of cells with higher HGF gene expression rather than a receptor of functional significance. In this role, targeting cells based on CD30 surface protein did result in enrichment of a more HGF^+^ population of cells, and helped to identify several additional markers which may represent a path forward towards more effective enrichment strategies (**Figures 6A, B**). We originally chose CD30 because of its limited expression by HGF^-^ cells (**Figure 4C**). Based on gene expression levels, some of the newly identified marker candidates such as CD320 and HMMR have moderate to high expression in HGF^-^ cells, but the expression level is higher in HGF^+^ cells. It could be the case that targeting this type of marker results in more efficient enrichment of HGF^+^ cells, but this requires further testing. Failing that, CD70 and ICAM1 are newly identified potential markers which have similar expression levels as CD30. Collectively, the markers of HGF^+^ CD4^+^ T cells represent new tools to pursue improved enrichment of HGF^+^ cells, and there is potential to target the markers individually or in combination to achieve that goal. Highly enriched populations of HGF^+^ cells would enable bulk surface marker screening by flow cytometry and in-depth characterization of the gene expression of these cells.

While there is a gap in the literature regarding HGF producing T cells, T cell expression of the receptor for HGF, c-Met, has been well described. In mice, surface c-Met expression by CD8^+^ cytotoxic lymphocytes (CTLs) is a marker of higher cytotoxic capacity compared to c-Met^-^ CTLs, and ligation of c-Met by HGF reduced this capacity, representing a immunosuppressive function of HGF (53). Interaction of Th1 and CTLs has been shown to be important in the reactivation of CTLs in response to re-encounter with antigen, and it could be interesting to test if HGF has an effect on the outcome of reactivation (54). It has also been shown that mouse CD4^+^ T cells stimulated in the presence of HGF protein upregulate c-Met, CCR4, and CXCR3, which results in the cells trafficking to the heart and liver (55). Expression of c-Met by human CD4^+^ and CD8^+^ T cells has also been described, where again c-Met appeared to be a marker of inflammatory circulating T cells; in this case the cells were found at higher frequency in the blood of patients with adaptive cardiac inflammation (56). Together, these previous findings may represent an opportunity to explore whether these cells also produce HGF, which could function in an autocrine manner, and to characterize what functions HGF may have in this context.

Some of the effects attributed to c-Met expression include apoptosis, protection from apoptosis, and survival which are somewhat paradoxical but occur as a result of c-Met signal transduction that can activate PI3K-Akt, resulting in survival signals, as well as the fact that, in cells that express both c-Met and the death receptor Fas, c-Met physically sequesters Fas, resulting in protection from apoptosis that can occur due to spontaneous clustering of Fas, and through blockade of Fas from ligation by Fas ligand (FasL) (21, 57). Meanwhile, ligation of c-Met by HGF causes the release of sequestered Fas and increased susceptibility to Fas mediated apoptosis (57). A recent study by Chen et al. suggests that the same mechanisms described by Wang et al. are functional in CD8^+^ T cells isolated from the blood of healthy human donors (58). They show that *in vitro* culture of CD8^+^ T cells with a high concentration of HGF protein causes release of c-Met sequestered Fas, and then results in Fas mediated apoptosis over a 72-hour culturing period. These results point towards the interesting possibility that expression of c-Met by T cells could represent a mechanism that increases resistance to FasL. Meanwhile, in the context of our results, the expression of HGF by T cells could be a mechanism that increases the susceptibility of target cells to FasL.

Treatment of mouse macrophages with HGF protein has been shown to promote wound healing in skeletal muscle, as well as a shift from M1 gene expression towards M2 (42, 59). We sought to test if the phenotype, with respect to M1 or M2 specifically, of human blood derived monocytes that had been *in vitro* differentiated into macrophages would be affected by treatment with exogenous HGF protein during or after polarization of the cells into M1 or M2 macrophages. The result indicated that HGF had no effect on TNF or MRC1 gene expression, even though it has been shown that human macrophages express c-Met protein and our gene expression data indicated the same (60). Even so, we also conducted a donor matched co-culture experiment where unpolarized M0, M1, and M2 macrophages were incubated with Th0 or Th1-like cells and CD3/CD28 stimulatory beads. qPCR data from the material left in the wells after removal of the T cells and washing steps indicated that the type of T cell added to the well had no effect on TNF or MRC1 expression. On the other hand, although unexpected, these variables did affect HGF gene expression, where co-culture of M2 and Th1 cells had the highest expression. This experiment suggested that T cell and macrophage co-culture may warrant further study with respect to HGF, because there could be an interaction which increases HGF gene expression in either macrophages, T cells, or both.

In conclusion, we have shown that *in vitro* differentiated human Th1-like cells are positive for HGF mRNA and can release HGF protein. Of the Th-like subsets that were generated, Th1-like cells were significantly more positive for HGF protein than the other groups (**Figure 2C**; **Supplemental Table 2**). In terms of HGF gene expression, Th1-like cells were positive for HGF mRNA, while the other groups had several negative samples (**Figures 2A, D**). Therefore, statistical tests to compare these groups to Th1-like cells were not conducted. Enrichment of HGF positive cells from a pool of Th1-like cells was possible by performing positive selection based on surface CD30 protein expression. Inhibition of mTOR by treatment with PP242 was shown to suppress HGF levels, while treatment with the Akt inhibitor triciribine resulted in a significant increase. These drugs may be useful tools for further study of the signal transduction pathways involved in HGF production. In addition, we developed and described a method for conducting an HGF ELISpot, which we hope can aid the study of HGF producing cells in general. We hypothesize that production of HGF by human T cells could be an important factor for consideration in cell-cell interactions as well as in disease where Th1 cells are present.

## Data availability statement

The original contributions presented in the study are publicly available. This data can be found here: 10.6084/m9.figshare.22745666 and 10.6084/m9.figshare.22745762 (figshare).

## Ethics statement

Informed, written consent was obtained from the blood donors at the Department of Clinical Immunology, University Hospital Rigshospitalet, Copenhagen, and donated material was used without the possibility to identify case specific information.

## Author contributions

SF, AW, TB, and CN designed the research. SF performed the experiments, analyzed and interpreted the data, made the figures, and wrote the original draft of the paper. TB prepared the single-cell RNA-sequencing raw data for analysis. SF and TB performed the single-cell RNA-sequencing data analysis. SF, AW, TB, CN, CG, CB, and NØ revised and edited the manuscript. AW conceptualized the study and acquired the funding. All authors have read and agreed to the published version of the manuscript.
