# Supplementary material for: In vitro differentiated human CD4+ T cells produce hepatocyte growth factor
Source: Front Immunol. 2023 Jul 13;14:1210836. doi: 10.3389/fimmu.2023.1210836 (PMC10374024; doi:10.3389/fimmu.2023.1210836)
Supplement: Supplementary file 4 [file DataSheet_1.pdf]

Supplementary Material

Human $CD4^+$ T Cells Produce Hepatocyte Growth Factor

Shayne Lavondua Ford[1], Terkild Brink Buus[1], Claudia Nastasi[2], Carsten Geisler[1], Charlotte Menné Bonefeld[1], Niels Ødum[1], Anders Woetmann[1*]

*** Correspondence:** Anders Woetmann, LEO Foundation Skin Immunology Research Center, Department of Immunology and Microbiology, University of Copenhagen, Panum Institute, The Maersk tower, 07.12.76, Blegdamsvej 3B, DK-2200, Copenhagen, Denmark. E-mail: awoetmann@sund.ku.dk

1.1 Supplementary Figures

Supplemental figure 1. PSO-2, MF2059, and HGF ELISpot information. (**A**) HGF ELISpot. The wells of column 2 have been treated with 100 ng/mL SEA. Row 1: MF2059 5x10^{5} cells/well (negative control). Row 2: PSO-2 6.25x10^{4} cells/well (positive control). Row 3: PBMCs 1x10^{6} cells/well. Treatment of PBMCs with staphylococcal enterotoxin superantigen A resulted in detection of spots, indicating that T cell receptor (TCR)-major histocompatibility complex II (MHC-II) bridging stimulated the release of HGF protein (Li et al., 1999). Note that the marks on the right of the wells are due to pipet tips touching the PVDF membrane during washing steps. (**B**) The results of several HGF ELISA tests which included supernatant from 5x10^{5} PSO-2 cells incubated in media for 24 hours. The purpose of this graph is to give an idea of how much HGF protein PSO-2 produces, and also how variable the amount can be due to batch effects. This is why the data is transformed into percent of control in other graphs.

Supplemental figure 2. (**A**) Kinetics of CD45RO and CD45RA surface protein expression of the isolated naive $CD4^+$ T cells as they undergo *in vitro* differentiation towards a Th1-like phenotype. This

is the gating strategy after gating $CD3^+CD4^+$ cells that is behind the data that is summarized in figure 3B. Small debris, doublets, and dead cells have been gated out prior to the $CD3^+CD4^+$ gate. (**B**) Kinetics of HGF and Th1 gene expression in isolated $CD4^+$ Memory T cells that were treated with the same differentiation and testing conditions as the naive $CD4^+$ T cells in Figure 3C. (**C**) Th1-like and Th2-like cells were generated from 5 donors through 5-day *in vitro* differentiation followed by a 24-hour restimulation period with anti-CD3/CD28 beads at a ratio of 1 bead per 8 cells, and then tested for characteristic Th1 (TBX21, IFNG) and Th2 (GATA3, IL5) gene expression by qPCR. Statistics shown in graph (**C**) are the result of a two-way ANOVA with Šídák's multiple comparisons test to compare Th1 to Th2 in each gene group. P values: ns ($P > 0.05$), * ($P \leq 0.05$), ** ($P \leq 0.01$), *** ($P \leq 0.001$), **** ($P \leq 0.0001$).

Supplemental figure 3. Unstimulated PSO-2 cells were sorted using a BD FACSAria II into two fractions: $CD96^-CD52^-CD9^+CD30^{low}$ (CD9+) and $CD96^-CD52^-CD9^-CD30^+$ (CD30+). These fractions were tested for HGF in comparison to unsorted PSO-2 cells from a culture flask by (**A**) HGF ELISA of supernatant collected after 24-hour incubation. (**B**) qPCR for HGF mRNA. (**C**) Flow cytometry of normal PSO-2 cells stained for markers of interest. Debris, doublets, and dead cells were gated out of the results. (**D**) Th1-like cells were generated from naive $CD4^+$ T cells over a 5-day differentiation period and then left unstimulated (top panel) or were stimulated with anti-CD3/CD28 beads at a ratio of 1 bead per 8 cells in base media for 24 hours. The cells were treated with monensin during the last 4 hours of the 24-hour stimulation period. After this period, the beads were removed, and then the cells were stained for surface and intracellular markers. The plot shows IFN-γ on the y-axis and CD30 on the x-axis. Doublets and dead cells were excluded from the results and the $CD3^+CD4^+$ cell population was selected.

Supplemental figure 4. Differential gene expression in HGF^+ cells (**A**) Heatmap of the top 200 upregulated genes in HGF^+ versus HGF^- cells (avg_log2FC ≥ 0.283672). (**B**) Heatmap of downregulated genes in HGF^+ versus HGF^- cells (avg_log2FC ≤ -0.25102). Alternately, see this data in supplemental table 3.

Supplemental figure 5. (**A-B**) Study of HGF kinetics in PSO-2 when the cells are treated with 500 ng Ionomycin and 20 ng PMA. Matched vehicle control and treated cells were harvested and pelleted at the listed timepoints. The supernatant was tested via HGF ELISA (**A**), and material from the pellets by qPCR (**B**). (**C**) HGF ELISA of PSO-2 cells treated with several concentrations of rapamycin for 24 hours. (**D**) HGF qPCR of PSO-2 cells treated with several concentrations of rapamycin for 24 hours. (**E**) Naive $CD8^+$ T cells were purified from the blood of healthy donors and then treated with Th1 *in vitro* differentiation media for five days. Following that, $1x10^6$ cells per well were incubated in an HGF ELISpot in media, vehicle (DMSO), with anti-CD3/CD28 beads (ratio 3 beads to 5 cells), beads in combination with the Akt inhibitor API-2, or beads in combination with the mTORC1/C2 inhibitor PP242 for 24 hours. The resultant spots were counted and are presented as number of spots in the graph. Statistics in (**C-D**) are the result of a one-way ANOVA with Dunnett's multiple comparisons test. P values: ns ($P > 0.05$), * ($P \leq 0.05$), ** ($P \leq 0.01$), *** ($P \leq 0.001$), **** ($P \leq 0.0001$).

Supplemental table 1. Viability of Th-like cells from 7 donors after 5 days of differentiation in their respective media types. CD3/CD28 beads were removed, cells were counted, and viability assessed using Trypan blue and a BioRad TC20 cell counter.

Supplemental table 2. Extended data from Tukey's multiple comparisons test of all of the groups in figure 2C.

Supplemental table 3. Supplemental figure 4 heatmap data in table format for easier viewing.
