# Supplementary figures and images for: In vitro differentiated human CD4+ T cells produce hepatocyte growth factor
Source: Front Immunol. 2023 Jul 13;14:1210836. doi: 10.3389/fimmu.2023.1210836 (PMC10374024; doi:10.3389/fimmu.2023.1210836)

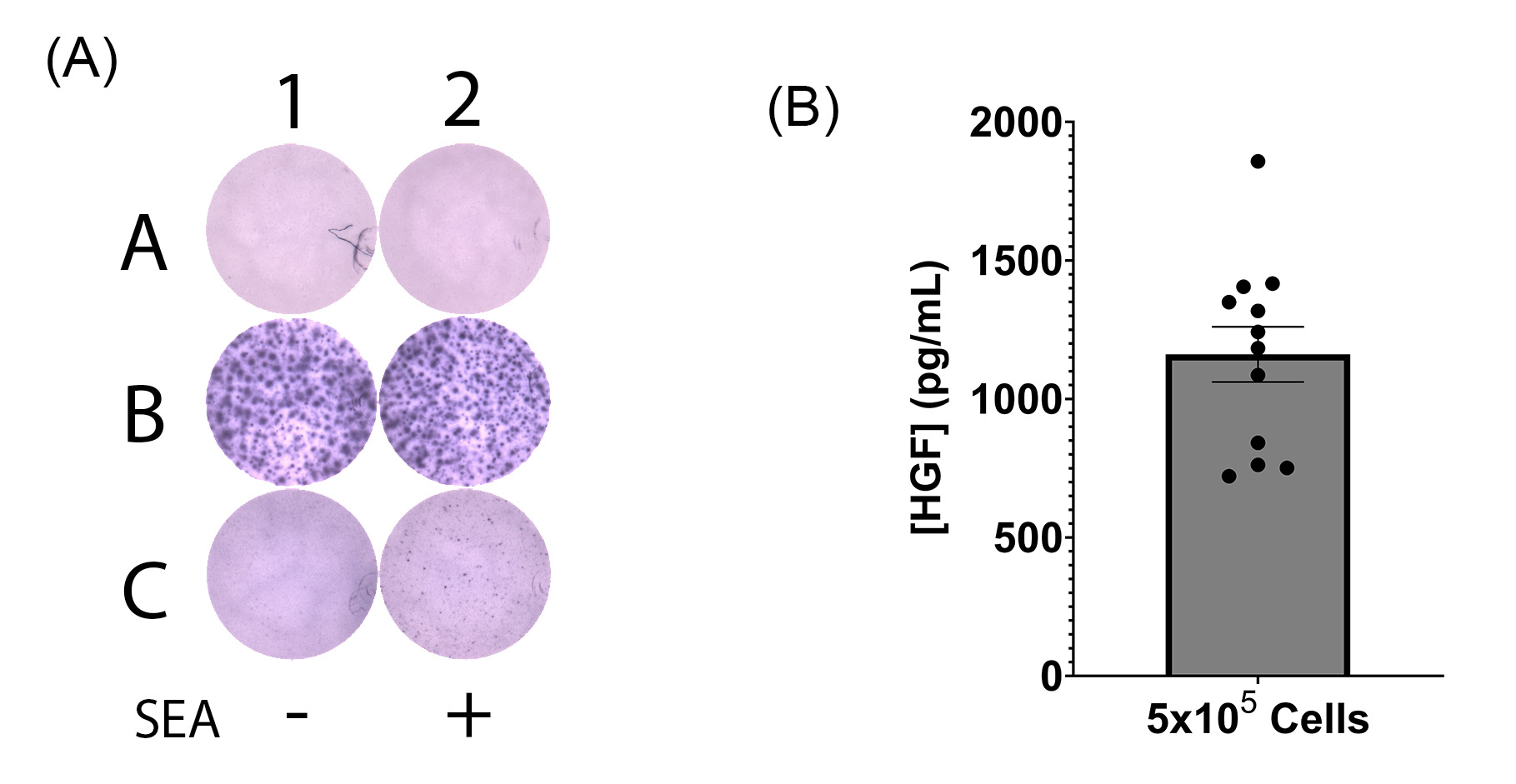

Supplement: Supplementary file 5 [file Image_1.jpeg]

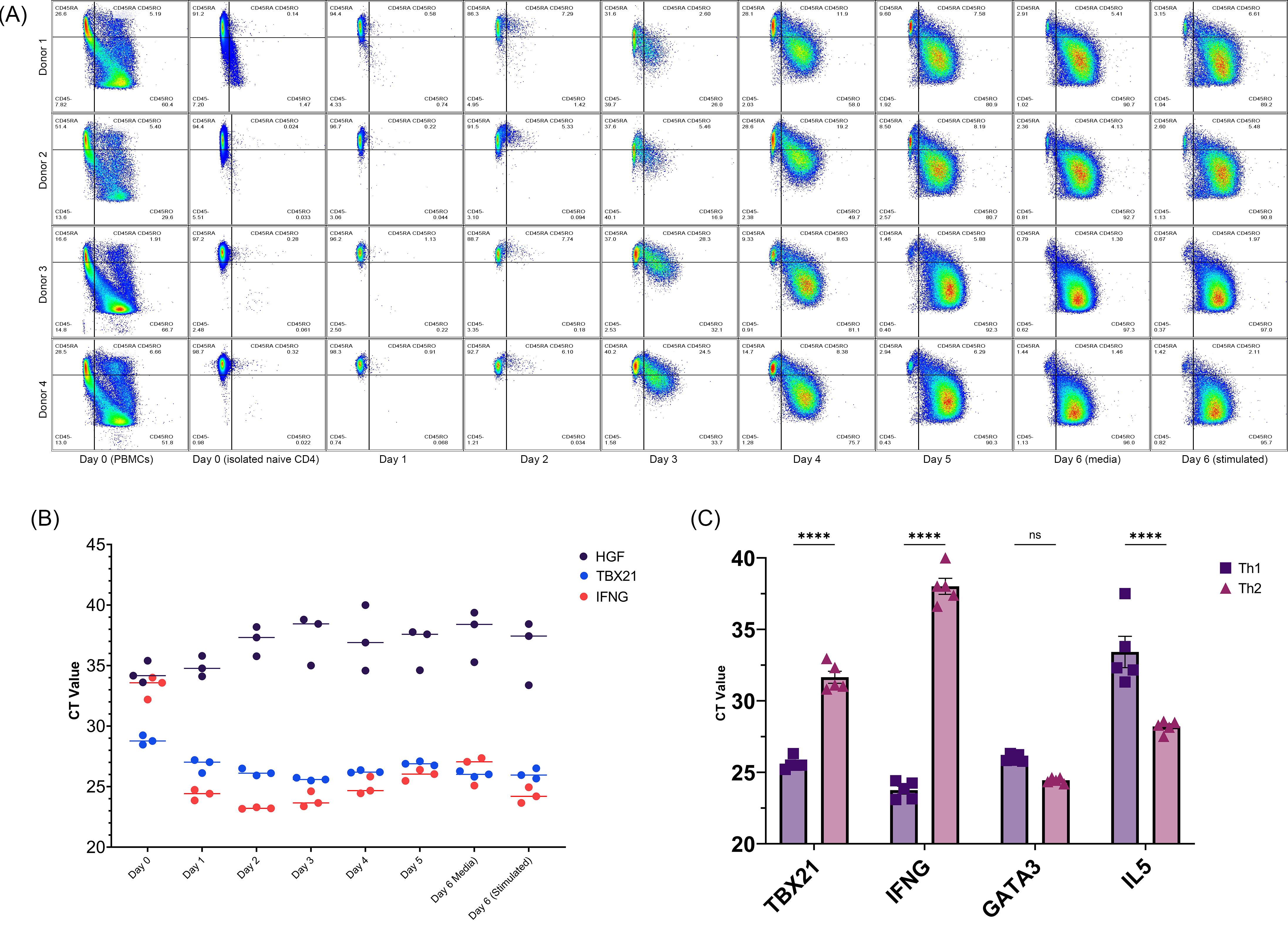

Supplement: Supplementary file 6 [file Image_2.jpeg]

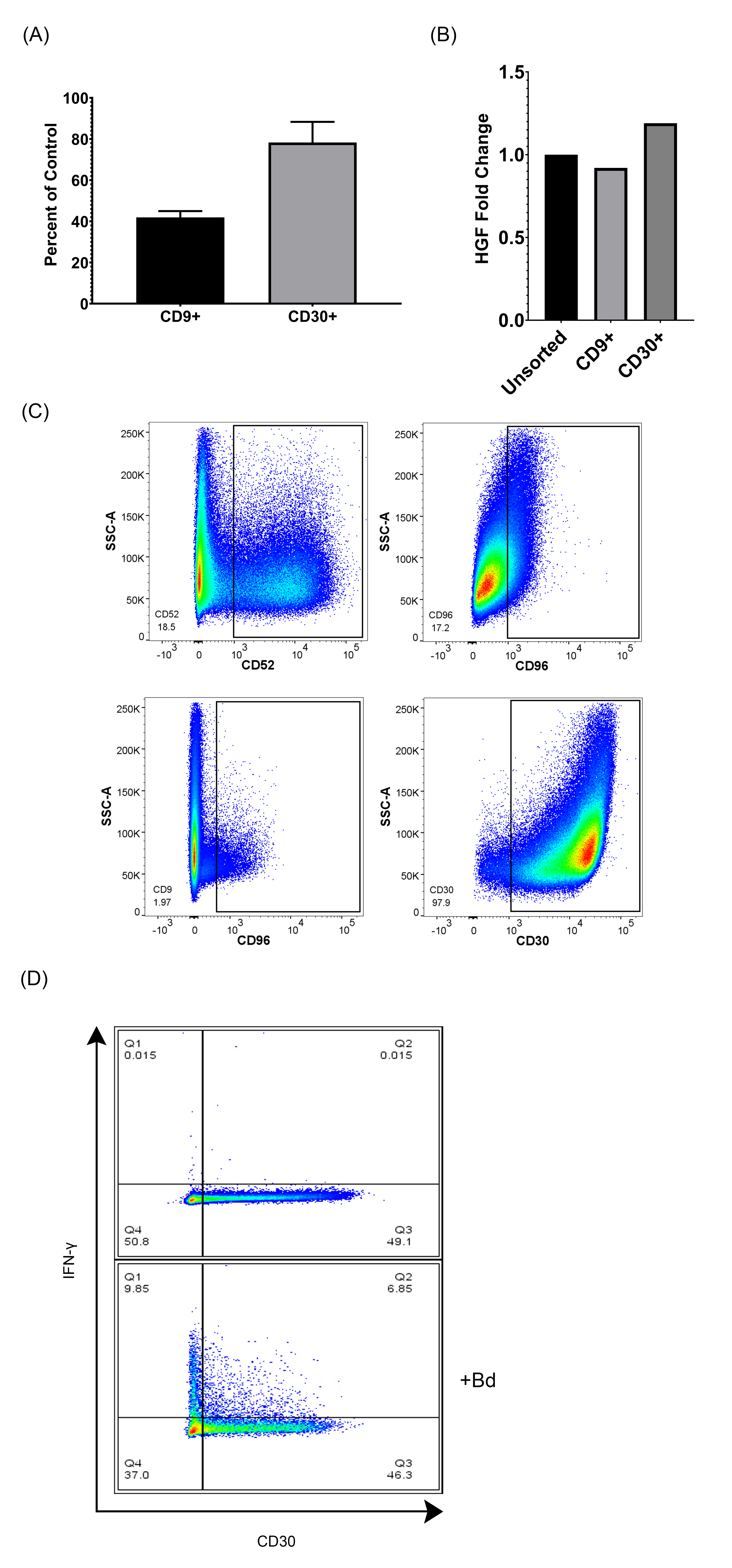

Supplement: Supplementary file 7 [file Image_3.jpeg]

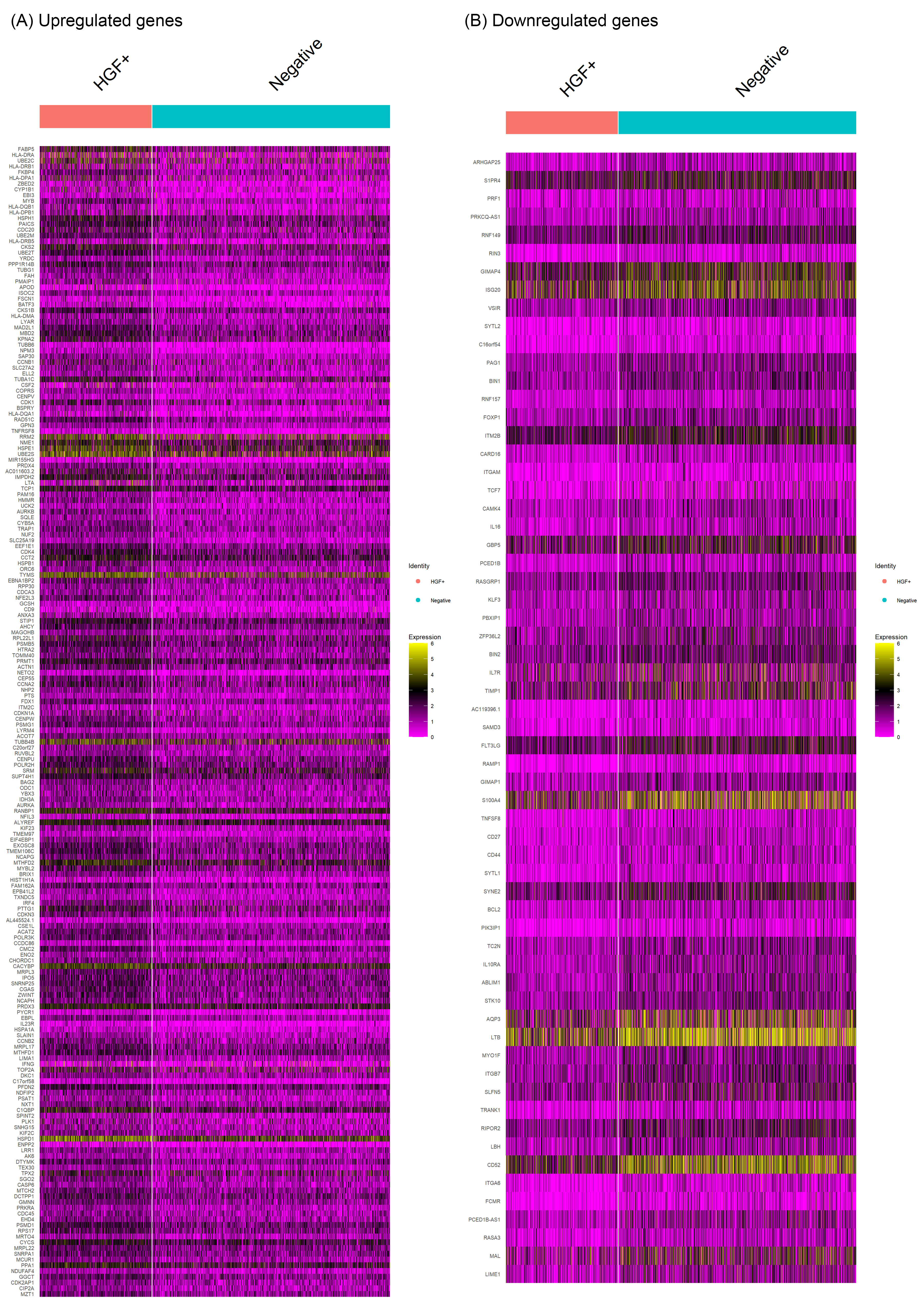

Supplement: Supplementary file 8 [file Image_4.jpeg]

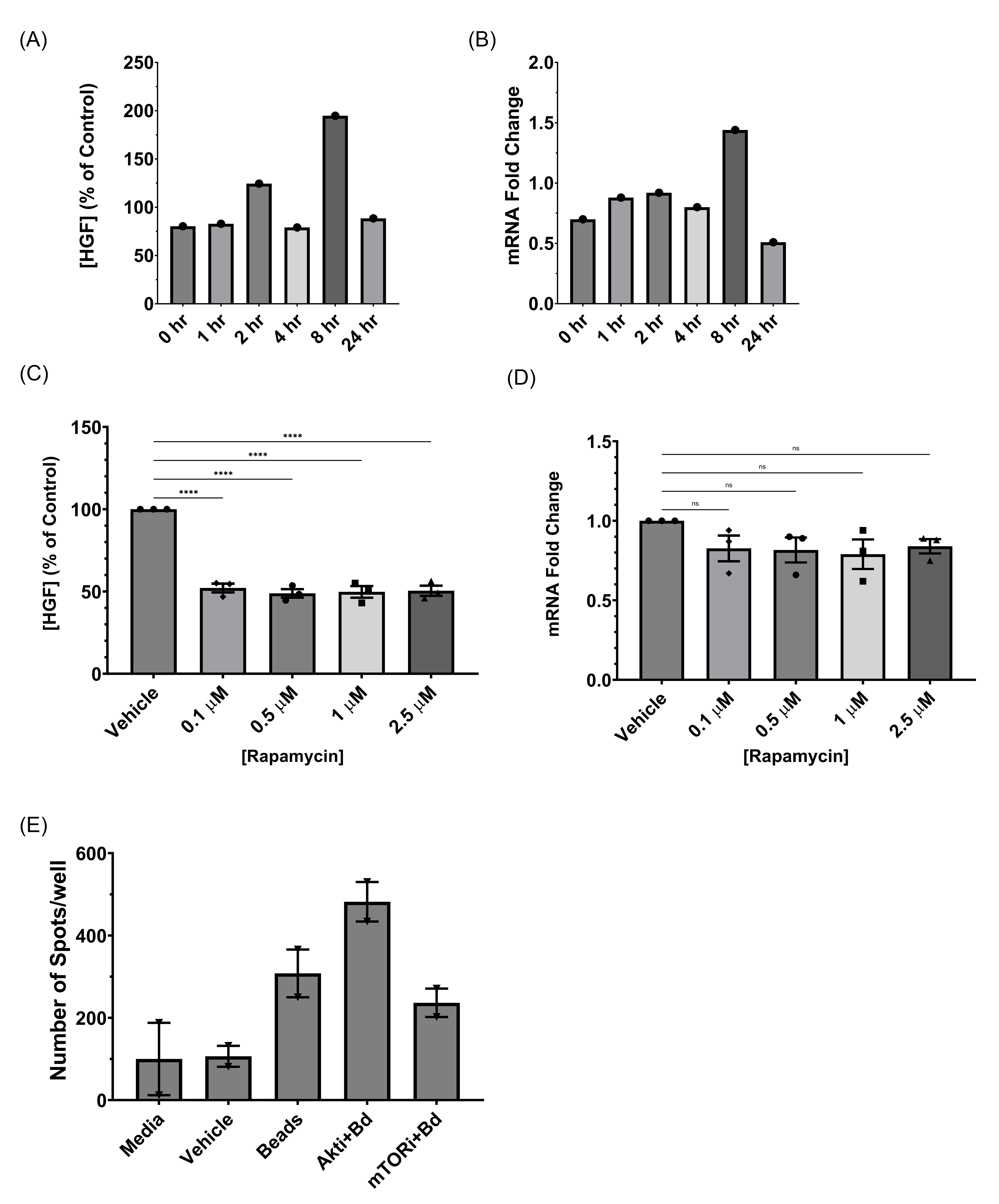

Supplement: Supplementary file 9 [file Image_5.jpeg]
